# Downregulation of exosomal miR-192-5p and miR-204-5p in subjects with nonclassic apparent mineralocorticoid excess

**DOI:** 10.1186/s12967-019-02143-8

**Published:** 2019-11-27

**Authors:** Alejandra Tapia-Castillo, Dominic Guanzon, Carlos Palma, Andrew Lai, Eric Barros, Fidel Allende, Andrea Vecchiola, Carlos E. Fardella, Carlos Salomón, Cristian A. Carvajal

**Affiliations:** 1 Department of Endocrinology, School of Medicine, Pontificia Universidad Católica de Chile, Diagonal Paraguay 362, piso 4, Santiago, 8330077 Chile; 2 Centro Traslacional de Endocrinología (CETREN-UC), Pontificia Universidad Católica de Chile, Santiago, Chile; 3 Millennium Institute on Immunology and Immunotherapy (IMII-ICM), Santiago, Chile; 4 Exosome Biology Laboratory, Centre for Clinical Diagnostics, University of Queensland Centre for Clinical Research, Royal Brisbane and Women’s Hospital, The University of Queensland, Brisbane, QLD 4029 Australia; 5 Department of Clinical Laboratories, School of Medicine, Pontificia Universidad Católica de Chile, Santiago, Chile; 6 Department of Clinical Biochemistry and Immunology, Faculty of Pharmacy, University of Concepción, Concepción, Chile

**Keywords:** Nonclassic AME, Mineralocorticoid receptor, MicroRNA, Exosomes, Hypertension

## Abstract

**Background:**

The “nonclassic” apparent mineralocorticoid excess (NC-AME) has been identified in approximately 7% of general population. This phenotype is characterized by low plasma renin activity (PRA), high serum cortisol (F) to cortisone (E) ratio, low cortisone, high Fractional Excretion of potassium (FEK) and normal-elevated systolic blood pressure (SBP). An early detection and/or identification of novel biomarkers of this phenotype could avoid the progression or future complications leading to arterial hypertension. Isolation of extracellular vesicles, such as exosomes, in specific biofluids support the identification of tissue-specific RNA and miRNA, which may be useful as novel biomarkers. Our aim was to identify miRNAs within urinary exosomes associated to the NC-AME phenotype.

**Methods:**

We perform a cross-sectional study in a primary care cohort of 127 Chilean subjects. We measured BP, serum cortisol, cortisone, aldosterone, PRA. According to the previous reported, a subgroup of subjects was classified as NC-AME (n = 10). Urinary exosomes were isolated and miRNA cargo was sequenced by Illumina-NextSeq-500.

**Results:**

We found that NC-AME subjects had lower cortisone (p < 0.0001), higher F/E ratio (p < 0.0001), lower serum potassium (p = 0.009) and higher FEK 24 h (p = 0.03) than controls. We found miR-204-5p (fold-change = 0.115; p 0.001) and miR-192-5p (fold-change = 0.246; p 0.03) are both significantly downregulated in NC-AME. miR-192-5p expression was correlated with PRA (r = 0.45; p 0.028) and miR-204-5p expression with SBP (r = − 0.48, p 0.027) and F/E ratio (r = − 0.48; p 0.026).

**Conclusions:**

These findings could support a potential role of these miRNAs as regulators and novel biomarkers of the NC-AME phenotype.

## Introduction

Arterial hypertension (AH) currently affects approximately 40% of adults worldwide and is a complex, multifactorial disease. This disease affects many metabolic systems in its pathogenesis, mainly the renal, vascular, and endocrine systems. Approximately 15% of essential hypertensive patients may be associated with endocrine pathologies related to mineralocorticoid receptor (MR) activation, such as primary aldosteronism and deficits in 11β-hydroxysteroid dehydrogenase type 2 (11β-HSD2) enzyme activity. Severe deficiency in 11β-HSD2 is also known as apparent mineralocorticoid excess (AME) and results from inefficient metabolism of cortisol (F) to its inactive metabolite cortisone (E), leading to the activation of the mineralocorticoid pathway by cortisol [1–3].

Recently, our group described the existence of a milder form of AME (nonclassic AME or NC-AME), characterized by an increased serum cortisol to cortisone ratio and low serum cortisone associated with normal-elevated systolic blood pressure (BP), and MR activation (detected by lower renin and a higher urinary potassium excretion rate) [4], suggesting the existence of a distinctive phenotypical spectrum in these subjects, which may be prevalent in a primary care cohort. In this study, we did not identify a genetic cause associated to that phenotype, which led us to hypothesize that epigenetic modifications (e.g. miRNAs affecting MR pathway genes) might be responsible for the onset and progression of hypertension of this phenotype, the nonclassic AME.

miRNAs are small noncoding RNA molecules that are approximately 21 to 23 bp long and they regulate target mRNAs through either translational repression, mRNA destabilization or a combination of both mechanisms. A single miRNA can regulate hundreds of genes, and collectively, miRNAs may regulate approximately 50–60% of the total transcriptome [5, 6]. The miRNAs influence gene expression both within their parental cells and promote intercellular communication by being transferred to other cells through nanovesicles—called exosomes—where they regulate cellular processes in the recipient cell [7–9]. Exosomes are small extracellular vesicles (EVs) with a size of 50–150 nm originating from endosomes. They are released from all cell types with a specific cargo (RNA, lipids and protein). Exosome cargo may mirror the physiological state or metabolic change of the cells of origin [10, 11]. Exosomes are isolated from different biofluids by ultracentrifugation (UCF) technology and could be potential biomarkers by itself or by their cargo for a variety of pathophysiological conditions, such as arterial hypertension [12–14].

The identification of miRNAs, RNA or proteins within exosomes associated to metabolic changes could be very informative of the local cellular physiology and pathophysiology. Thus, different translational approaches have been developed to identify specific miRNAs and proteins that are associated with pathophysiological conditions. The aim of the current study was to study the miRNA profile obtained from urinary exosomes from subjects with the nonclassic AME (NC-AME) phenotype.

## Methods

### Subjects

A total of 396 Chilean subjects between 10 and 65 years old and both genders were invited to participate from two primary care centers in Santiago, Chile. All subjects have a similar socioeconomic status and ethnicity, and they declared had not ingested licorice. For the current study, we excluded subjects with pre-puberal stage (Tanner < 2), kidney disease, uncontrolled diabetes mellitus, liver failure and heart failure. Subjects using glucocorticoids, oral contraceptives or spironolactone were excluded due to the known effects these have on adrenal hormones levels in plasma. Subjects with previously diagnosed secondary causes of hypertension, such as familial hyperaldosteronism, classic apparent mineralocorticoid excess (AME), hypercortisolism, and renovascular disease, were also not included in this study. Subjects with glucocorticoid remediable aldosteronism (GRA) (with positive molecular diagnosis), primary aldosteronism (PA) (aldosterone-renin ratio > 25 and PRA < 1 ng/ml*h) or potential hypercortisolism (urinary free cortisol > 110 μg/24 h [15]) were also excluded from this study. Patients who were using antihypertensive drugs that affect the renin–angiotensin–aldosterone system (RAAS), such as beta-blockers, angiotensin-converting enzyme (ACE) inhibitors, angiotensin II receptor blockers, and diuretics, or those who received amlodipine or doxazosin for at least 4 weeks for blood pressure control due to the neutral effect over the RAAS were also excluded from this study. The protocol followed in this study was in agreement with the guidelines of the Declaration of Helsinki and was approved by the Ethical Committee of the Faculty of Medicine, Pontificia Universidad Catolica de Chile (CEC-MEDUC#14–268). A written informed consent was signed by all participants.

### Clinical characteristics

All subjects underwent a complete physical examination performed by trained endocrinologists of the Pontificia Universidad Catolica de Chile. The subjects’ heights were measured using a wall-mounted Harpenden stadiometer (Holtain). Three BP measurements were obtained from the right arm at consecutive 5-minute intervals using an oscillometric method (Dinamap CARESCAPE V100, GE Healthcare, Medical Systems Information Technologies, Milwaukee, WI) with the subjects in a seated position. Hypertension was diagnosed when blood pressure was higher than 130/80 mm Hg in adults [16] and adolescents [17] after at least 2 different measurements.

### Biochemical assays

After overnight fasting, basal blood samples were obtained between 08:00 and 10:00 AM. Serum aldosterone (SA) and plasma renin activity (PRA) were measured by radioimmunoassay using a commercial kit (Coat-A-Count Kit; Siemens, Los Angeles, CA and DiaSorin, Stillwater, MN, respectively). At the same time, spot and 24-h urine samples were collected. Serum and urinary cortisol and cortisone were quantified using LC–MS/MS and levels were validated according to the parameters suggested by the Food and Drug Administration (FDA) and Clinical and Laboratory Standard Institute (CLSI) using deuterated internal standards of cortisol and cortisone (cortisol-d4 and cortisone-d2) in an Agilent 1200 series HPLC equipment coupled to an ABSciex 4500-QTrap mass spectrometer.

### Identification of NC-AME subjects

After exclusion criteria were applied, we perform the study with 127 subjects (age 10–65 years old). In all subjects we analyzed serum cortisone and the cortisol to cortisone ratio (F/E) [4]. Subjects with both cortisone lower than percentile 25th and a serum cortisol to cortisone ratio higher than percentile 75th were classified as subjects suspected of nonclassic AME (NC-AME) [4]. We identify 10 subjects with NC-AME suspicion, corresponding to the 7.8% of studied subjects (Table 1). We performed a cross-sectional study in NC-AME subjects compared with a control group (N = 15 subjects) similar in age, gender, body mass index and urinary sodium excretion (Table 1).

**Table 1:** Clinical and biochemical characteristics of the studied subjects

Clinical and biochemical parameters	NC-AME	Controls
n	10	15
Female, %	60% (6/10)	53.3% (8/15)
Age, years	24.4 [12.3–42.7]	25.8 [12.1–47.13]
BMI, kg/m^2^	27.3 [22.0–28.9]	24.6 [21.0–28.9]
SBP, mmHg	126.5 [110.3–154.1]	124.3 [108.0–143.3]
DBP, mmHg	73.8 [66.6–84.4]	78.0 [68.3–85.0]
Aldosterone, ng/dl	8.9 [4.7–13.5]	12.7 [7.4–15.6]
PRA, ng/ml*h	1.2 [0.7–2.2]	2.1 [1.5–3.5]
Serum K^+^, mEq/l	4.0 [3.8–4.4]	4.5 [4.1–4.8]*
Serum Cortisol, µg/dl	12.5 [9.4–13.8]	8.1 [7.8–12.1]
Serum Cortisone, µg/dl	1.9 [1.8–2.1]	3.0 [2.5–3.1]*
Serum F/E ratio	6.0 [4.6–6.7]	3.1 [2.3–4.2]*
FEK 24 h %	7.9 [6.0–9.0]	5.9 [4.7–7.2]*
Urinary sodium excretion, mEq/24 h	138.5 [54.5–215.8]	128.0 [71.0–199.0]
Urinary potassium excretion, mEq/24 h	49.0 [35.8–72.3]	53.0 [35.0–60.0]
Urinary Na^+^/K^+^ ratio	2.1 [1.4–3.7]	2.6 [1.9–3.8]
TTKG	5.6 [4.4–7.2]	4.4 [3.5–4.9]
Urinary F/E ratio	0.5 [0.4–0.5]	0.3 [0.3–0.4]*
(THF + allo-THF)/THE ratio	0.7 [0.6–0.8]	0.6 [0.5–0.9]

Values correspond to median [Q1–Q3]

*BMI* body mass index, *SBP* systolic blood pressure, *DBP* diastolic blood pressure, *PRA* plasma renin activity, *K*^*+*^ potassium, *F* cortisol, *E* cortisone, *FEK* fractional excretion of potassium, *Na*^*+*^ sodium, *TTKG* trans-tubular potassium gradient, *THF* tetrahydrocortisol; *THE* tetrahydrocortisone

* p < 0.05, Mann–Whitney test

### Exosomes isolation

Approximately 13 ml of spot urine was collected from each subject in the morning (08:00–10:00 AM). Urine was storage at − 80 °C with protease inhibitor cocktail (Roche) until analyses. Urine was centrifuged at 4 °C at 1000×*g* for 15 min to eliminate the cell debris. The supernatant was centrifuged at 17,000×*g* for 15 min at 4 °C. The supernatant was then filtered with a 0.22 μm filter and ultracentrifuged at 200,000×*g* for 1 h at 4 °C in a ultracentrifuge Thermo-Sorvall WX80+ (Thermo Fisher Scientific Inc., Asheville, NC, USA) with a TH-660 swinging bucket rotor (K factor = 82.6). Exosome pellets were resuspended in 100 μl of PBS.

### Electron microscopy

Exosomal shape and size was determined by transmission electron microscopy (TEM). For this, 15 μl of an exosome pellet was added onto a carbon-coated cooper grid (300 mesh) for 1 min and stained with 2% uranyl acetate for 1 min. Grids were visualized at 80 kV in a Phillips Tecnai transmission electron microscope.

### Nanoparticle Tracking Analysis (NTA)

NTA measurements were performed using a NanoSight NS500 instrument (Malvern, UK) with the NanoSight NTA 3.0 nanoparticle tracking and analysis software (Version Build 0064) as previously described [18]. Three videos of 30 s were processed and analyzed. A minimum of 200 completed tracks per video was collected for each analyzed sample. NTA post-acquisition settings were optimized and kept constant between samples and each video was then analyzed to determine the mean, mode, and median particle size, together with an estimated number of particles per ml of plasma. A spreadsheet (Excel, Microsoft Corp., Redmond, Washington) was automatically generated, recording the concentration of each particle size. 100 nm polystyrene latex microspheres (Malvern NTA 4088) were routinely analyzed to confirm the instrument performance.

### Western blot analysis of exosome markers

Characteristic exosomal markers were determined using western blot analysis. Exosomes were resuspended in RIPA buffer (ThermoFisher Scientific, USA) in order to extract total protein and the protein concentration was determined using the bicinchoninic acid method [BCA Protein Assay kit (Thermofisher, Scientific Inc., Asheville, NC, USA)]. Protein lysates (20–50 μg) were separated by polyacrylamide gel electrophoresis (SDS-PAGE), transferred to nitrocellulose membranes (Bio-Rad, USA), and blocked with 5% skim milk in phosphate buffered saline containing 0.1% Tween-20 (PBST). Next, membranes were probed with a primary mouse monoclonal antibody anti-CD63 (1:200; sc-5275; Santa Cruz Bio-technology) and a primary rabbit monoclonal antibody anti-TSG101 (1:10.000; ab125011; Abcam, USA), followed by incubation with horseradish peroxidase-conjugated goat anti-rabbit IgG-HRP (1:10.000; ab6939; Abcam, USA) or rabbit anti-mouse IgG-HRP antibodies (1:10.000; ab6728; Abcam, USA). Proteins were detected using enhanced chemiluminescence (ECL Western Blotting substrate reagent, Pierce, USA).

### Exosomal RNA isolation

RNA was isolated by organic extraction using the Trizol^®^ reagent according to the manufacturer’s instructions. The SPECTROstar Nano Microplate Reader (BMG LABTECH) spectrophotometer was used to quantify the RNA concentration. Following a cleanliness check and blank measurement using RNase-free water, 2 μL of each sample was pipetted on to a microdrop well on an LVis plate. The RNA concentration was measured using the MARS Data Analysis microplate reader software.

### Next generation sequencing

Sequencing libraries were generated using the TruSeq 1 Small-RNA Library Prep Kit (Illumina, San Diego, Ca, USA) according to manufacturer’s instructions. A total of 100 to 300 ng of exosomal RNA was used as input for library preparation. These RNA samples were barcoded by ligation with unique adaptor sequences to allow pooling of samples into groups of 24. Subsequently, these ligated samples were reverse transcribed, PCR amplified, and size selected using gel electrophoresis. Finally, DNA libraries were eluted from the extracted gel pieces overnight in 200 μL nuclease free water. The elution, containing the pooled DNA library, was further processed for cluster generation using the NextSeq 500 high output kit for 75 cycles and sequencing using the Illumina NextSeq 500 sequencing platform.

### Identification of miRNAs in raw sequencing data

Initially, raw FASTQ files were processed to remove barcode and adaptor sequences. Subsequently, these files were analyzed using the miRDeep2 program to identify known miRNAs [19]. The miRDeep2 algorithm requires a genomic index and miRNA database to perform analysis. The human genome (version 19) prebuilt index was obtained from the bowtie website (http://bowtie-bio.sourceforge.net/index.shtml). The miRNA reference database (version 20) was obtained from the miRBase website (http://www.mirbase.org/) [20]. Sequencing data have been deposited in the GEO database with the accession number GSE138556.

### Taqman quantitative real-time PCR assay

To validate the RNA sequencing data, we performed a Taqman qRT-PCR analysis. Reverse transcription was performed for each specific miRNA using the Taqman MicroRNA RT kit (Applied Biosystems, Foster City, CA). The expression of the RNU6 snRNA was used as an internal normalization control. The expression levels of the miRNAs were evaluated with the TaqMan MicroRNA Assay kit in the RotorGene 6000 thermocycler (Corbett Research, Sydney, Australia).

### Bioinformatic analyses

Gene target identification for identified miRNAs was performed using the miRwalk software. Candidate miRNAs identified from the sequencing data were imported into miRwalk. A total of 4 miRNA gene target databases (miRWalk, TargetScan, miRanda, and RNA22) were provided to miRwalk for analysis. Subsequently, gene targets were filtered to identify those RNAs that are targeted by the same miRNA within at least two separate databases. From this selection, genes shown to be regulated by at least two miRNAs were extracted and subjected to gene ontology analysis. MirPath v.3 analysis of downregulated miRNAs (Diana Tools Software) was performed to find pathways in KEGG and the gene-ontology (GO) enrichment analyses (Additional file 1).

### Statistical analysis

Data are presented as percentages for categorical variables. Variables with a non-normal distribution are reported as median and interquartile ranges [Q1–Q3]. In situations where a variable was not normally distributed, a bootstrapping procedure with 1000 iterations was performed. We evaluated the differences between NC-AME subjects and control subjects by t-test with bootstrapping in non-normal variables. A 2-sided *p* value of < 0.05 was considered to be statistically significant. We also perform univariate and multivariate regression analyses, using stepwise selection, to find predictive variables of miRNA expression. All analyses were performed using SPSS 20 and the GraphPad Prism v5.0 software.

Differential expression and statistical analysis of sequencing data was performed by the DESeq 2 package in R [21]. This package uses a generalized linear model to perform differential expression. Statistical analysis and significance were calculated using a Wald test and adjusted for multiple testing using the Benjamini and Hochberg procedure.

## Results

### Clinical and biochemical analyses

From the 127 subjects studied, we identified 10 subjects that fulfilled the criteria for NC-AME (7.8%), which were compared with 15 control subjects matched by age, gender and BMI. The NC-AME group included 5 adolescents and 5 adults, and the control group included 7 adolescents and 8 adults. Baseline characteristics of both groups are shown in Table 1. NC-AME subjects had lower serum cortisone (1.9 [1.8–2.1] vs 2.9 [2.5–3.1] μg/dl; p < 0.0001), lower serum potassium (4.0 [3.8–4.4] vs 4.5 [4.1–4.8] mEq/l; p = 0.009), and a higher serum cortisol to cortisone ratio (6.0 [4.6–6.7] vs 3.1 [2.3–4.2]; p < 0.0001) and FEK 24% (7.9 [5.9–9.0] vs 5.9 [4.7–7.2]; p = 0.03) than the control group. PRA trend to be lower in NC-AME than in control subjects (1.2 [0.7–2.2] vs 2.1 [1.5–3.5] ng/ml*h; p = 0.05).

### Exosome isolation and characterization

**Fig. 1:**
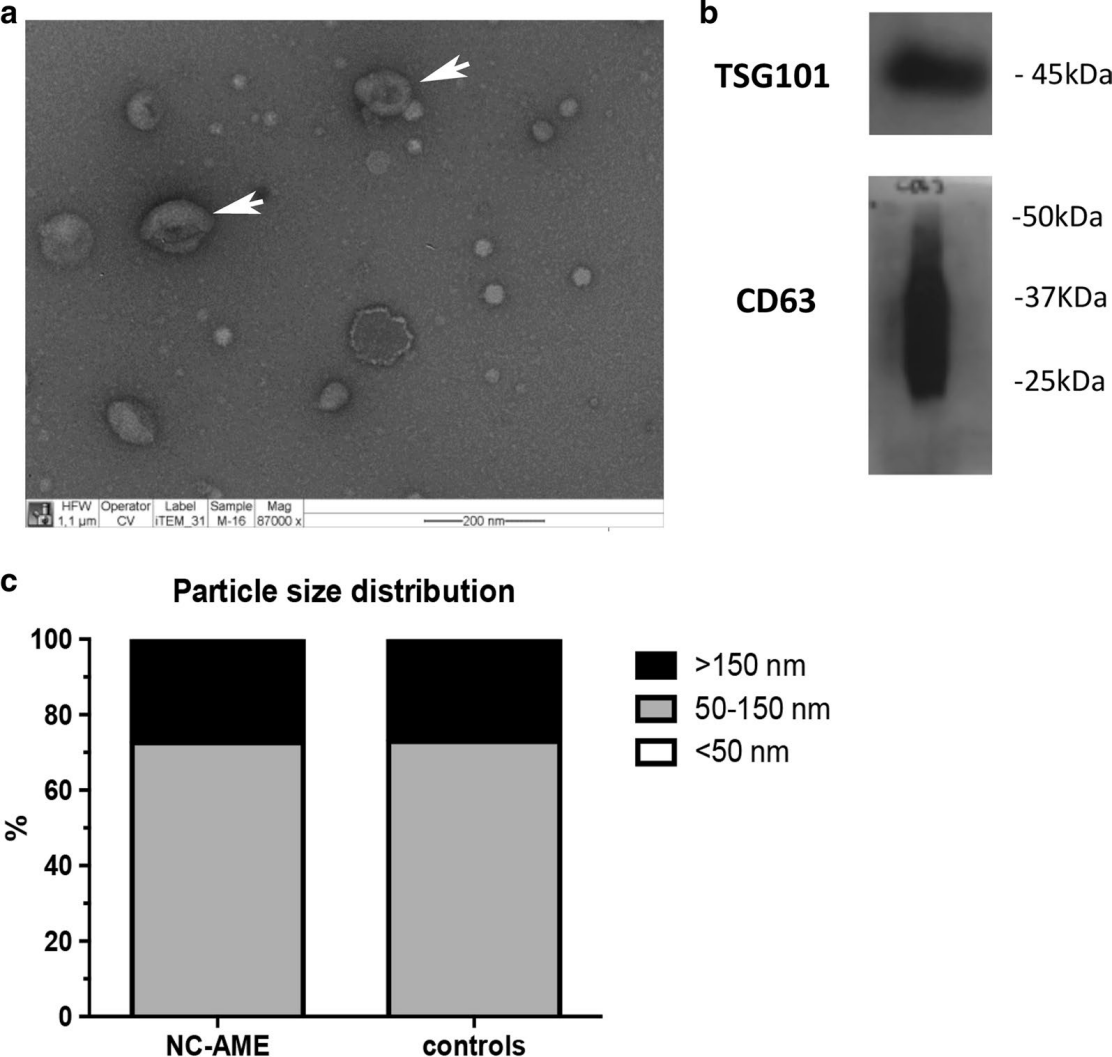
Characterization of urinary exosomes. **a** Identification of exosomes by Transmission Electron Microscopy (TEM) (indicated by white arrows). **b** Western blot of exosomal proteins (TSG101 and CD63). **c** Percentages of particles in 3 size ranges (< 50 nm; 50–150 nm; > 150 nm)

The morphological characteristics of the urinary exosomes (i.e. round donut shape and 50–150 nm diameter) were observed by transmission electron microscopy (TEM) and are shown in Fig. 1a. The presence of the known exosome markers CD63 and TSG101 were confirmed by immunoblot analysis as shown in Fig. 1b.

We found that 73% of the urinary nanovesicles with a size between 50 and 150 nm in both groups of subjects (NC-AME and controls) and 27% corresponded to nanovesicles with a size greater than 150 nm (Fig. 1c). The percentage of nanovesicles smaller than 50 nm was less than 1%.

**Fig. 2:**
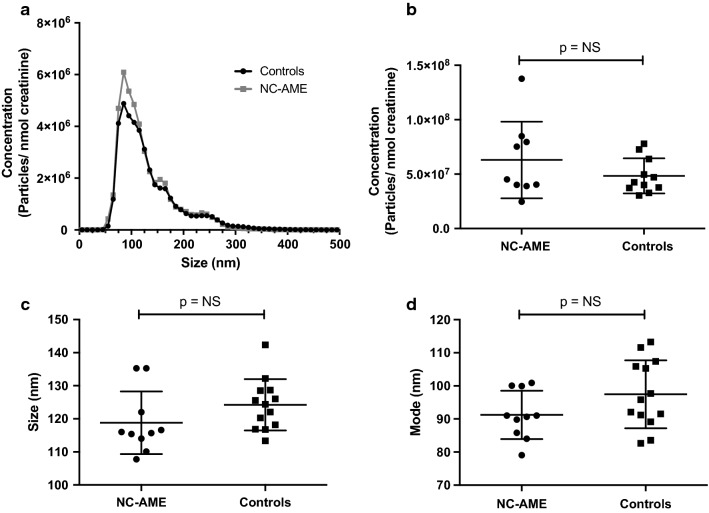
Characteristics of urinary exosomes in NC-AME subjects and control groups. **a** Size and concentration of urinary exosomes (Black circles, control subjects; Grey squares, NC-AME subjects). **b** Total concentration of exosomes obtained of spot urine and normalized by creatinine. **c** Urinary exosomes size, **d** mode of urinary exosomes in NC-AME and controls subjects

The exosome concentration was normalized to urinary creatinine and after normalization, we did not observe significant differences between NC-AME subject and control subjects (6.3 × 10^7^ ± 3.5 × 10^7^ vs 4.8 × 10^7^ ± 1.6 × 10^7^ particles/nmol creatinine, p = 0.2) (Fig. 2a, b). The size of urinary exosomes did not show statically differences in NC-AME subjects compared to control subjects, determined either by the mean (118.8 ± 9.5 nm vs 124.2 ± 7.8 nm; p = 0.06) (Fig. 2c) or the mode (91.2 ± 7.3 nm vs 97.5 ± 10.3 nm; p = 0.12) (Fig. 2d).

### Urinary exosome microRNA profiling and data analysis

**Fig. 3:**
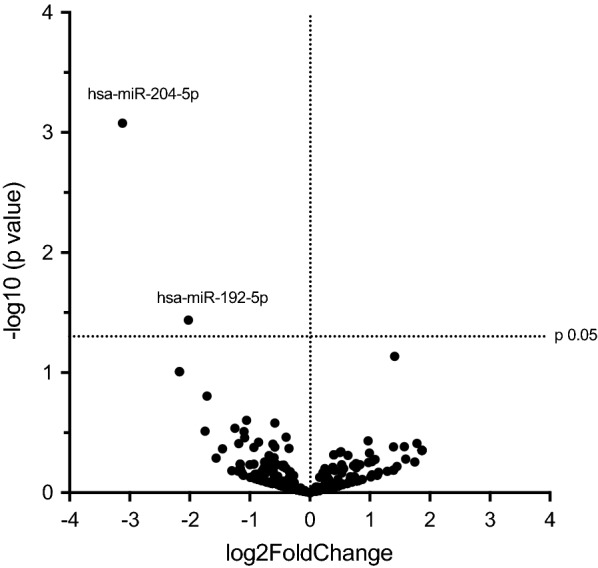
A volcano plot showing differential expression of miRNAs in NC-AME subjects vs. control subjects. Comparisons of all miRNAs assessed in RNA-seq analysis of miRNA isolated from urinary exosome of NC-AME or healthy control subjects. The volcano plot displays the relationship between fold-change and significance between the two groups using a scatter plot view. The X-axis is the Log2 of miRNA expression (fold change) levels between subjects with NC-AME and control subjects. The Y-axis adjusts the p value as a function of − Log10

To identify the expression profile of miRNA in urinary exosomes, we used a high-throughput sequencing technology, RNA-sequencing. From the 2822 described miRNAs, we detected 355 miRNAs in urinary exosomes, of which 170 miRNAs showed a fold-change higher than 1 (or Log2 > 0) and 185 miRNAs showed a fold-change lower than 1 (or Log2 < 0) in NC-AME compared to control subjects. However, only two miRNAs were found to be significantly downregulated (p < 0.05). Statistical analyses indicated that the expression of hsa-miR-204-5p (fold change = 0.115; p < 0.05) and hsa-miR-192-5p (fold change = 0.246; p < 0.05) were downregulated in NC-AME subjects as compared to the control group (Table 2, Fig. 3).

**Table 2:** Exosomal urinary miRNA expression observed in NC-AME versus control subjects

MiRNA	Fold change	p value
*hsa*-*miR*-*204*-*5p*	0.1149	0.0008*
*hsa*-*miR*-*192*-*5p*	0.2462	0.0366*
*hsa*-*let*-*7i*-*5p*	2.6600	0.0731
*hsa*-*miR*-*125b*-*1*-*5p*	0.2218	0.0983
*hsa*-*miR*-*125b*-*2*-*5p*	0.2218	0.0983
*hsa*-*miR*-*200b*-*3p*	0.3050	0.1569
*hsa*-*miR*-*125a*-*5p*	0.4817	0.2497
*hsa*-*miR*-*203a*-*3p*	0.6677	0.2626
*hsa*-*miR*-*29a*-*3p*	0.4214	0.2910
*hsa*-*let*-*7d*-*3p*	0.2981	0.3070
*hsa*-*miR*-*28*-*3p*	0.4679	0.3101
*hsa*-*miR*-*27a*-*3p*	0.7597	0.3445
*hsa*-*miR*-*222*-*3p*	0.4708	0.3489
*hsa*-*miR*-*7977*	1.9583	0.3696
*hsa*-*miR*-*99b*-*5p*	0.5530	0.3794
*hsa*-*miR*-*769*-*5p*	3.4432	0.3881
*hsa*-*miR*-*501*-*3p*	0.4408	0.3898
*hsa*-*miR*-*92a*-*1*-*3p*	0.6523	0.3945
*hsa*-*miR*-*183*-*5p*	2.9774	0.4137
*hsa*-*miR*-*21*-*3p*	2.6293	0.4153
*hsa*-*miR*-*92a*-*2*-*3p*	0.6673	0.4165
*hsa*-*miR*-*29c*-*3p*	0.5239	0.4203
*hsa*-*miR*-*27b*-*3p*	0.7858	0.4271
*hsa*-*miR*-*345*-*5p*	0.3645	0.4313

* miRNAs expression is shown as fold-change. A p value lower than 0.05 is considered significant

### Validation of miRNAs by Taqman qRT-PCR

**Fig. 4:**
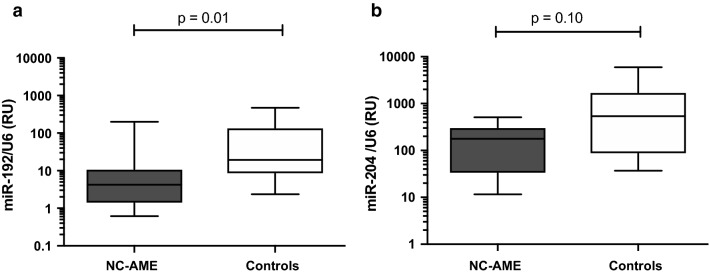
miRNA expression by Taqman qRT-PCR in urinary exosomes. The expression levels of **a** miR-192-5p, **b** miR-204-5p in urinary exosomes from NC-AME and healthy control subjects were validated by real-time RT-PCR analysis, shown in scatted plot. Mean and standard deviation are shown. The p value was obtained by Mann–Whitney test. *RU* relative units

Taqman RT-qPCR analyses were performed to confirm and validate the significant down-regulated miRNAs (miR-192-5p and miR-204-5p) observed by RNA-seq in urinary exosomes samples of NC-AME group (Fig. 3). miR-192-5p expression is lower (4.3 [1.9–11.5] vs 19.3 [8.3–137.6] RU; p = 0.01) in NC-AME vs controls subjects (Fig. 4a). These results were consistent with the high-throughput sequencing analysis. miR-204-5p expression showed a trend to be downregulated in NC-AME vs controls subjects (177.6 [32.8–308.6] vs 540.3 [85.3–1728] RU; p = 0.10) (Fig. 4b).

### Bioinformatic studies for target prediction of miR-192-5p and miR-204-5p

Bioinformatics analyses were performed using four databases for target prediction (miRWalk, TargetScan, miRanda, and RNA22). We identified 212 RNA targets for mir-192-5p and 902 RNA targets for miR-204-5p. We found that both miRNAs target the RNA of ATP1A2 gene (ATPase Na+/K+ Transporting Subunit Alpha 2). MIR-192-5p is predicted to target the RNA of ARHGAP1 (Rho GTPase Activating Protein) and CUL3 (Cullin 3) genes, among others. MIR-204-5p was found to target RNA of ATP1B4 (ATPase Na^+^/K^+^ Transporting Family Member Beta 4), ATP2B4 (ATPase Plasma Membrane Ca^2+^ Transporting 4), NEDD4 (E3 Ubiquitin-Protein Ligase NEDD4), NR3C1 (Glucocorticoid Receptor), NR3C2 (Mineralocorticoid Receptor), YWHAG (14-3-3 Protein Gamma), ARHGAP30 (Rho GTPase Activating Protein), ARHGEF26 (Rho Guanine Nucleotide Exchange Factor (GEF), ARHGEF37 (Rho Guanine Nucleotide Exchange Factor (GEF) and WNK3 (Serine/Threonine-Protein Kinase WNK3) genes.

### Association of miRNAs and clinical-biochemical parameters

Association studies of miRNA expression and clinical and biochemical characteristics of all subjects showed that hsa-miR-192-5p expression correlates positively with PRA (r = 0.45; p = 0.028) and hsa-miR-204-5p expression displayed an inverse correlation with SBP (r = − 0.48, p = 0.027) and serum cortisol to cortisone ratio (r = − 0.48; p = 0.026) (Table 3). A linear regression supports a model where the expression of hsa-miR-204-5p is predicted by DBP, serum cortisol to cortisone ratio, FEK, and gender (R^2^ = 0.86). Similarly, multivariate analyses support a model where expression of miR-192-5p can be predicted by serum aldosterone levels and urinary sodium excretion (R^2^ = 0.37).

**Table 3:** Association between miRNA expression and clinical and biochemical parameters in all subjects

	miR-204-5p		miR-192-5p	
	r	p	r	p
Age, years	− 0.26	0.26	− 0.4	0.07
BMI, kg/m^2^	− 0.33	0.14	− 0.21	0.36
SBP, mmHg	− 0.48	0.027*	− 0.24	0.31
DBP, mmHg	− 0.36	0.11	− 0.31	0.18
PRA, ng/ml*h	0.14	0.56	0.45	0.03
Cortisol, μg/dl	− 0.43	0.05	− 0.28	0.21
Cortisone, μg/dl	0.42	0.06	− 0.1	0.7
Serum F/E ratio	− 0.48	0.02*	− 0.18	0.4
Urinary F/E ratio	− 0.53	0.03*	− 0.27	0.25
(THF + allo-THF)/THE ratio	− 0.17	0.49	− 0.12	0.57

Pearson correlation with bootstrapping

*BMI* body mass index, *SBP* systolic blood pressure, *DBP* diastolic blood pressure, *PRA* plasma renin activity, *F* cortisol, *E* cortisone, *THF* tetrahydrocortisol, *THE* tetrahydrocortisone

* A p value lower than 0.05 is considered significant

## Discussion

We identified 355 miRNAs in urinary exosomes, of which only 2 were significant downregulated in NC-AME subjects compared with controls (Table 2, Fig. 3). By Taqman RT-qPCR we confirmed that miR-192-5p was downregulated, meanwhile miR-204-5p have a trend to a lower expression in NC-AME subjects.

Previous studies indicate that miR-192-5p and miR-204-5p are highly expressed in the kidney [22, 23]. MIR-192 is expressed at higher levels in the renal cortex than in the medulla [24] and is 20-fold higher in the proximal tubules than in the glomeruli. miR-192 is involved in regulation of sodium transport in renal epithelial cells [25]. A recent study by Baker et al. showed low expression of miR-192-5p in kidney biopsy specimens from patients with hypertensive nephrosclerosis and hypertension [26]. Reduced expression of miR-192-5p is associated with an increase in Na/K-ATPase function (ATP1B1 gene), which contributes to hypertension and kidney injury [26]. Similarly, it has been shown that loss of miR-192-5p is associated with fibrogenesis in diabetic nephropathy [27]. All of these examples highlight the role of miR-192-5p in the renal system, which could be useful as a biomarker for some types of kidney diseases, especially in AH. Bioinformatics studies shown that miR-192-5p could regulate genes related to both small GTPase mediated signal transduction (CUL3, ARHGAP1, ARHGAP36, ARHGEF39) and sodium transport (ATP1A2, SCL5A12), which have been previously related to the mineralocorticoid receptor [28–31] and sodium/potassium exchange [26, 32, 33] pathways (Table 4), suggesting a role in the etiopathogenesis of arterial hypertension.

**Table 4:** Target genes of miR-204-5p and 192-5p and its predicted renal and global effect

miRNA (downregulated in NC-AME)	Target gene	Affected pathway	Predicted renal effect	Global effect	References
miR-204-5p	NR3C2 NEDD4-2 YWHAG (14-3-3 protein gamma) ATP1A4 ATP1B1 DNMT3a	ENaC channel activity; NCC symporter activity. Na^+^/K^+^ exchange DNA Methylation	Increased renal Na^+^ reabsorption Increased promoter methylation of HSD11B2 gene	Increase plasma volume (low renin); High BP. Decreased cortisol to cortisone metabolism; High F/E ratio	[33] [34, 40–42] [36–38]
miR-192-5p	ATP1A1 ATP1A2 RAC1 ARHGAP ARHGEF	Na^+^/K^+^ exchange Sodium reabsorption independent of aldosterone	Increased renal Na^+^ reabsorption	Increase plasma volume (low renin); high BP	[25] [26] [28–31]

*NC-AME* nonclassical AME, *BP* blood pressure

The miR-204-5p is also highly expressed in kidney tissues and has been shown to be downregulated in advanced diabetic nephropathy biopsies [34]. Other studies have observed a reduction of miR-204-5p expression in epithelial cells associated with reduced expression of claudins 10, 16 and 19, suggesting a critical, albeit indirect, a role of this miRNA in maintaining epithelial cell function [35]. Using mirWalk, we found that miR-204-5p could potentially regulate genes downstream MR-activation related to sodium transmembrane transport (NEDD4, ATP1A2, ATP2B4, WNK3), cellular response to hormone stimuli (NEDD4, ATP1A2, NR3C1, NR3C2, YWHAG), and genes that regulate of molecular function in the cell (ATP2B4, ATP1A2, NEDD4, WNK3, ARHGEF37, ARHGEF26, ARHGAP30, YWHAG), suggesting a potential role for miR-204-5p in renal pathways associated with sodium/potassium exchange (Table 4). Recently, has been reported that miR-204 is a critical regulator of de novo DNA methylation, through affecting the DNA methyltransferase 3-alpha (DNMT3a) [36]. In this way, we speculate that low expression of miR-204 seen in NC-AME, could be associated with higher expression of DNMT3a and hypermethylation the HSD11B2 promoter [37, 38], decreasing the HSD1B2 expression, and lately affecting the cortisol to cortisone metabolism (Table 4).

Our results also show that both miR-192-5p and miR-204-5p could regulate ATP1A2 expression and it has been previously shown that this α2-isoform of the Na/K-ATPase pump mediates ouabain-induced hypertension in mice and increased vascular contractility in vitro [33]. Association studies indicated that miR-192-5p expression is correlated with PRA which suggest being a potential biomarker of MR activation, also can be predicted by aldosterone and sodium urinary excretion, which is in agreement with a previous report by Elvira-Matelot et al. that showed renal miR-192-5p expression is decreased by aldosterone infusion [39]. Similarly, hsa-miR-204-5p expression was negatively associated with serum cortisol to cortisone and SBP, which highlights it as a potential biomarker in NC-AME subjects. However, further research should be performed to validate these miRNAs as potential biomarker of NC-AME and regulator of key genes in MR pathway [34, 40–42].

To the best of our knowledge, this is the first study addressing the role of urinary exosomes and their miRNA content in subjects with nonclassic AME. We found that urinary exosomes from NC-AME subjects have lower expression of two miRNAs compared with controls subjects. Nevertheless, there are a few limitations in our study. First, the sample size of patients and controls was relatively small, and these findings should be validated in larger cohorts to better evaluate the sensitivity and specificity of miR-204-5p and miR-192-5p as potential biomarkers of nonclassic AME. Second, the predicted miRNA gene targets that were identified using bioinformatics methods would need to be validated in vitro, and the role of these miRNAs in the regulation of cellular pathways will need to be further investigated.

## Conclusions

The present study showed a lower expression of miR-192-5p and miR-204-5p in urinary exosomes from NC-AME compared with control subjects, which are associated with low PRA and high cortisol to cortisone ratio. Moreover, our results shown that NC-AME is also present in normotensive subjects (Table 1), which open new biomedical challenges aimed to identification of novel and early biomarkers, preventive actions and also potential second hits associated to NC-AME phenotype.

We suggest these miRNAs may have a potential role as early biomarkers and may regulators of the mineralocorticoid activity in NC-AME subjects, which will be useful to may uncover and understand the mechanisms associated to this phenotype.

## Supplementary information

**Additional file 1: Figure S1.** A heatmap plot and dendrogram of predicted KEGG pathways obtained by MirPath v.3 analysis of hsa-miR-204-5p and hsa-miR-192-5p. **Figure S2.** A heatmap plot and dendrogram of gene-ontology (GO) enrichment analysis with of hsa-miR-204-5p and hsa-miR-192-5p.

## Data Availability

The datasets used and/or analyzed during the current study are available from the corresponding author on reasonable request.

## Abbreviations

11β-HSD2: 11β-hydroxysteroid dehydrogenase type 2
ACE: angiotensin-converting enzyme
AH: arterial hypertension
AME: apparent mineralocorticoid excess
BP: blood pressure
E: cortisone
EVs: extracellular vesicles
F: cortisol
F/E: cortisol to cortisone ratio
FEK: fractional excretion of potassium
LC–MS/MS: liquid chromatography tandem–mass spectrometry
MR: mineralocorticoid receptor
NC-AME: nonclassic apparent mineralocorticoid excess
NTA: nanoparticle tracking analysis
PA: primary aldosteronism
PRA: plasma renin activity
RAAS: renin-angiotensin-aldosterone system
SA: serum aldosterone
TEM: transmission electron microscopy
UCF: ultracentrifugation

## References

[1] Campino C, Carvajal CA, Cornejo J, San Martin B, Olivieri O, Guidi G, Faccini G, Pasini F, Sateler J, Baudrand R (2010). 11beta-Hydroxysteroid dehydrogenase type-2 and type-1 (11beta-HSD2 and 11beta-HSD1) and 5beta-reductase activities in the pathogenia of essential hypertension. Endocrine.

[2] Myles K, Funder JW (1994). Type I (mineralocorticoid) receptors in the guinea pig. Am J Physiol.

[3] Ferrari P, Lovati E, Frey FJ (2000). The role of the 11beta-hydroxysteroid dehydrogenase type 2 in human hypertension. J Hypertens.

[4] Tapia-Castillo A, Baudrand R, Vaidya A, Campino C, Allende F, Valdivia C, Vecchiola A, Lagos CF, Fuentes CA, Solari S (2019). Clinical, biochemical, and genetic characteristics of “Nonclassic” apparent mineralocorticoid excess syndrome. J Clin Endocrinol Metab.

[5] Baek D, Villen J, Shin C, Camargo FD, Gygi SP, Bartel DP (2008). The impact of microRNAs on protein output. Nature.

[6] Krol J, Busskamp V, Markiewicz I, Stadler MB, Ribi S, Richter J, Duebel J, Bicker S, Fehling HJ, Schubeler D (2010). Characterizing light-regulated retinal microRNAs reveals rapid turnover as a common property of neuronal microRNAs. Cell.

[7] Yu X, Odenthal M, Fries JW (2016). Exosomes as miRNA carriers: formation-function-future. Int J Mol Sci.

[8] French KC, Antonyak MA, Cerione RA (2017). Extracellular vesicle docking at the cellular port: extracellular vesicle binding and uptake. Semin Cell Dev Biol.

[9] Das S, Halushka MK (2015). Extracellular vesicle microRNA transfer in cardiovascular disease. Cardiovasc Pathol.

[10] Camussi G, Deregibus MC, Bruno S, Grange C, Fonsato V, Tetta C (2011). Exosome/microvesicle-mediated epigenetic reprogramming of cells. Am J Cancer Res.

[11] Michael A, Bajracharya SD, Yuen PS, Zhou H, Star RA, Illei GG, Alevizos I (2010). Exosomes from human saliva as a source of microRNA biomarkers. Oral Dis.

[12] Romaine SP, Charchar FJ, Samani NJ, Tomaszewski M (2016). Circulating microRNAs and hypertension–from new insights into blood pressure regulation to biomarkers of cardiovascular risk. Curr Opin Pharmacol.

[13] Butterworth MB (2015). MicroRNAs and the regulation of aldosterone signaling in the kidney. Am J Physiol Cell Physiol.

[14] Bartel DP (2004). MicroRNAs: genomics, biogenesis, mechanism, and function. Cell.

[15] Nieman LK, Biller BM, Findling JW, Newell-Price J, Savage MO, Stewart PM, Montori VM (2008). The diagnosis of Cushing’s syndrome: an endocrine society clinical practice guideline. J Clin Endocrinol Metab.

[16] Whelton PK, Carey RM, Aronow WS, Casey DE, Collins KJ, Dennison Himmelfarb C, DePalma SM, Gidding S, Jamerson KA, Jones DW (2017). 2017 ACC/AHA/AAPA/ABC/ACPM/AGS/APhA/ASH/ASPC/NMA/PCNA guideline for the prevention, detection, evaluation, and management of high blood pressure in adults: a report of the American College of Cardiology/American Heart Association Task Force on Clinical Practice Guidelines. J Am Coll Cardiol.

[17] Flynn JT, Kaelber DC, Baker-Smith CM, Blowey D, Carroll AE, Daniels SR, de Ferranti SD, Dionne JM, Falkner B, Flinn SK (2017). Clinical practice guideline for screening and management of high blood pressure in children and adolescents. Pediatrics.

[18] Sarker S, Scholz-Romero K, Perez A, Illanes SE, Mitchell MD, Rice GE, Salomon C (2014). Placenta-derived exosomes continuously increase in maternal circulation over the first trimester of pregnancy. J Transl Med.

[19] Mackowiak SD (2011). Identification of novel and known miRNAs in deep-sequencing data with miRDeep2. Curr Protoc Bioinf.

[20] Kozomara A, Griffiths-Jones S (2014). miRBase: annotating high confidence microRNAs using deep sequencing data. Nucleic Acids Res.

[21] Love MI, Huber W, Anders S (2014). Moderated estimation of fold change and dispersion for RNA-seq data with DESeq2. Genome Biol.

[22] Gracia T, Wang X, Su Y, Norgett EE, Williams TL, Moreno P, Micklem G, Karet Frankl FE (2017). Urinary exosomes contain MicroRNAs capable of paracrine modulation of tubular transporters in kidney. Sci Rep.

[23] Sun Y, Koo S, White N, Peralta E, Esau C, Dean NM, Perera RJ (2004). Development of a micro-array to detect human and mouse microRNAs and characterization of expression in human organs. Nucleic Acids Res.

[24] Tian Z, Greene AS, Pietrusz JL, Matus IR, Liang M (2008). MicroRNA-target pairs in the rat kidney identified by microRNA microarray, proteomic, and bioinformatic analysis. Genome Res.

[25] Mladinov D, Liu Y, Mattson DL, Liang M (2013). MicroRNAs contribute to the maintenance of cell-type-specific physiological characteristics: miR-192 targets Na+/K+-ATPase beta1. Nucleic Acids Res.

[26] Baker MA, Wang F, Liu Y, Kriegel AJ, Geurts AM, Usa K, Xue H, Wang D, Kong Y, Liang M (2019). MiR-192-5p in the kidney protects against the development of hypertension. Hypertension.

[27] Ma X, Lu C, Lv C, Wu C, Wang Q (2016). The expression of miR-192 and its significance in diabetic nephropathy patients with different urine albumin creatinine ratio. J Diabetes Res.

[28] Nagase M, Fujita T (2013). Role of Rac1-mineralocorticoid-receptor signalling in renal and cardiac disease. Nat Rev Nephrol.

[29] Loirand G, Pacaud P (2014). Involvement of Rho GTPases and their regulators in the pathogenesis of hypertension. Small GTPases.

[30] Tapia-Castillo A, Carvajal CA, Campino C, Vecchiola A, Allende F, Solari S, Garcia L, Lavanderos S, Valdivia C, Fuentes C (2014). Polymorphisms in the RAC1 gene are associated with hypertension risk factors in a Chilean pediatric population. Am J Hypertens.

[31] Tapia-Castillo A, Carvajal CA, Campino C, Hill C, Allende F, Vecchiola A, Carrasco C, Bancalari R, Valdivia C, Lagos C (2015). The expression of RAC1 and mineralocorticoid pathway-dependent genes are associated with different responses to salt intake. Am J Hypertens.

[32] Kaplan JH (2005). The sodium pump and hypertension: a physiological role for the cardiac glycoside binding site of the Na, K-ATPase. Proc Natl Acad Sci U S A.

[33] Dostanic I, Paul RJ, Lorenz JN, Theriault S, Van Huysse JW, Lingrel JB (2005). The alpha2-isoform of Na-K-ATPase mediates ouabain-induced hypertension in mice and increased vascular contractility in vitro. Am J Physiol Heart Circ Physiol.

[34] Rudnicki M, Perco P, D’haene B, Leierer J, Heinzel A, Muhlberger I, Schweibert N, Sunzenauer J, Regele H, Kronbichler A (2016). Renal microRNA- and RNA-profiles in progressive chronic kidney disease. Eur J Clin Invest.

[35] Wang FE, Zhang C, Maminishkis A, Dong L, Zhi C, Li R, Zhao J, Majerciak V, Gaur AB, Chen S, Miller SS (2010). MicroRNA-204/211 alters epithelial physiology. FASEB J.

[36] Lin X, Xu F, Cui RR, Xiong D, Zhong JY, Zhu T, Li F, Wu F, Xie XB, Mao MZ (2018). Arterial calcification is regulated via an miR-204/DNMT3a regulatory circuit both in vitro and in female mice. Endocrinology.

[37] Friso S, Carvajal CA, Fardella CE, Olivieri O (2015). Epigenetics and arterial hypertension: the challenge of emerging evidence. Transl Res.

[38] Friso S, Pizzolo F, Choi SW, Guarini P, Castagna A, Ravagnani V, Carletto A, Pattini P, Corrocher R, Olivieri O (2008). Epigenetic control of 11 beta-hydroxysteroid dehydrogenase 2 gene promoter is related to human hypertension. Atherosclerosis.

[39] Elvira-Matelot E, Zhou XO, Farman N, Beaurain G, Henrion-Caude A, Hadchouel J, Jeunemaitre X (2010). Regulation of WNK1 expression by miR-192 and aldosterone. J Am Soc Nephrol.

[40] Potus F, Graydon C, Provencher S, Bonnet S (2014). Vascular remodeling process in pulmonary arterial hypertension, with focus on miR-204 and miR-126 (2013 Grover Conference series). Pulm Circ.

[41] Courboulin A, Paulin R, Giguere NJ, Saksouk N, Perreault T, Meloche J, Paquet ER, Biardel S, Provencher S, Cote J (2011). Role for miR-204 in human pulmonary arterial hypertension. J Exp Med.

[42] Yu Z, Zhan X (2018). Xia Li: miR-204 inhibits hypertension by regulating proliferation and apoptosis of vascular smooth muscle cells. Int J Clin Exp Med.

