# Supplementary material for: Downregulation of exosomal miR-192-5p and miR-204-5p in subjects with nonclassic apparent mineralocorticoid excess
Source: J Transl Med. 2019 Nov 27;17:392. doi: 10.1186/s12967-019-02143-8 (PMC6880399; doi:10.1186/s12967-019-02143-8)
Supplement: Supplementary file 1 — Additional file 1: Figure S1. A heatmap plot and dendrogram of predicted KEGG pathways obtained by MirPath v.3 analysis of hsa-miR-204-5p and hsa-miR-192-5p. Figure S2. A heatmap plot and dendrogram of gene-ontology (GO) enrichment analysis with of hsa-miR-204-5p and hsa-miR-192-5p. [file 12967_2019_2143_MOESM1_ESM.pdf]

ADDITIONAL DATA

Supplementary Figure 1.

Figure S1. Heatmap of predicted KEGG pathways

A heatmap plot and dendrogram shows the MirPath v.3 analysis of hsa-miR-204-5p and hsa-miR-192-5p (Diana Tools Software), which identifies 4 significant signaling pathways by KEGG analysis (Vasopressin-regulated water reabsorption (hsa04962), Neurotrophin signaling pathway (hsa04722), Focal adhesion (hsa04510), Regulation of actin cytoskeleton (hsa04810)), affected by these miRNAs. Vasopressin-regulated water reabsorption (hsa04962) was common for both miRNAs. The KEGG analyzes indicate that both miRNAs may play a role in other systems associated to hydro-electrolytic control as vasopressin-regulated water reabsorption (hsa04962), which strength the role of miR-204-5p and miR-192-5p in the renal physiology.

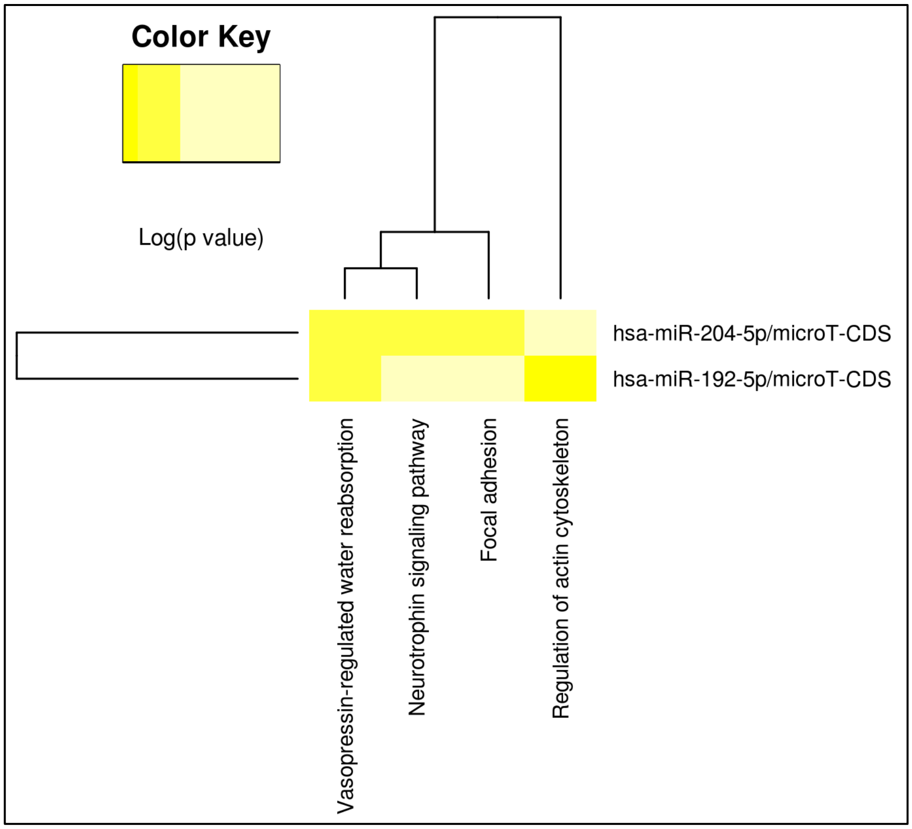

Supplementary Figure 2

Figure S2. Heatmap of gene-ontology (GO) enrichment analysis

A heatmap plot and dendrogram shows the gene-ontology (GO) enrichment analysis with of hsa-miR-204-5p and hsa-miR-192-5p. It identified several biological processes associated with ion binding (GO:0043167), cellular nitrogen compound metabolic process (GO:0034641), neurotrophin TRK receptor signaling pathway (GO:0048011), biosynthetic process (GO:0009058), organelle (GO:0043226), protein binding transcription factor activity (GO:0000988). A common biological process for both miRNA was ion binding (GO:0043167) which are in agreement with similar previous studies (Gracia et al., 2017).

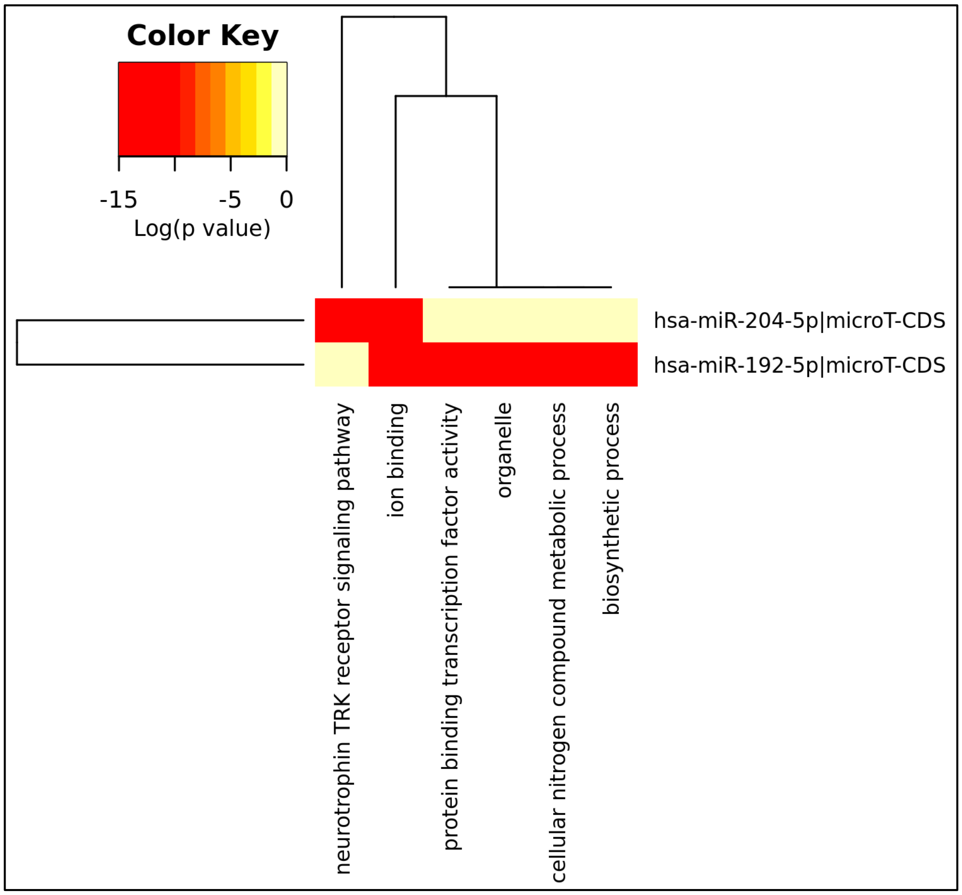

REFERENCES

Gracia, T., Wang, X., Su, Y., Norgett, E. E., Williams, T. L., Moreno, P., . . . Squires, P. E. (2017). Urinary Exosomes Contain MicroRNAs Capable of Paracrine Modulation of Tubular Transporters in Kidney. *Scientific Reports, 7*, 40601. doi:10.1038/srep40601
